# Expectations Modulate the Magnitude of Attentional Capture by Auditory Events

**DOI:** 10.1371/journal.pone.0048569

**Published:** 2012-11-07

**Authors:** Anatole Nöstl, John E. Marsh, Patrik Sörqvist

**Affiliations:** 1 Department of Building, Energy and Environmental Engineering, University of Gävle, Gävle, Sweden; 2 School of Psychology, University of Central Lancashire, Preston, United Kingdom; 3 Linnaeus Centre HEAD, Swedish Institute for Disability Research, Linköping University, Linköping, Sweden; University of Salamanca- Institute for Neuroscience of Castille and Leon and Medical School, Spain

## Abstract

What determines the magnitude of attentional capture by deviant sound events? We combined the cross-modal oddball distraction paradigm with sequence learning to address this question. Participants responded to visual targets, each preceded by tones that formed a repetitive cross-trial standard sequence. In Experiment 1, with the standard tone sequence …-660-440-660-880-… Hz, either the 440 Hz or the 880 Hz standard was occasionally replaced by one of two deviant tones (220 Hz and 1100 Hz), that either differed slightly (by 220 Hz) or markedly (by 660 Hz) from the replaced standard. In Experiment 2, with the standard tone sequence …-220-660-440-660-880-660-1100-… Hz, the 440 Hz and the 880 Hz standard was occasionally replaced by either a 220 Hz or a 1100 Hz pattern deviant. In both experiments, a high-pitch deviant was more captivating when it replaced a low-pitch standard, and a low-pitch deviant was more captivating when it replaced a high-pitch standard. These results indicate that the magnitude of attentional capture by deviant sound events depends on the discrepancy between the deviant event and the expected event, not on perceived local change.

## Introduction

Sound events that are novel in the present sound environment can capture attention [1], and divert it away from what is attended to, towards the novel stimulus and back again [2], resulting in a temporary, measureable, interruption to the focal activity [3]. The experiments reported here are concerned with how memory of past environmental stimulation is used to form expectations of what will happen in the sound environment and address the following question: Is the magnitude of attentional capture by deviant auditory events a function of the difference between (a) what we expect on the basis of what we have learned about the sound environment and (b) what actually happens in the sound environment?

The processes that underlie auditory attentional capture have been extensively studied in the cross-modal oddball distraction paradigm [3]. In this paradigm, participants view and respond to sequences of visual targets, and each target is preceded by a task-irrelevant background sound. The same sound is presented on most trials (a standard), but on rare trials, the standard is replaced by an infrequently presented sound (called a deviant or an oddball). When a deviant sound event is presented, response time to the target is typically increased (e.g., [4], [5]). This behavioral interruption–here refered to as an oddball effect–is accompanied with a specific brain response called mismatch negativity (MMN; an event-related potential component that seems to be elicited when the auditory system has detected an irregularity [6]), and seems to result from a comparison process that registers the difference between the neural representation of the deviation and some memory trace of the recent auditory past [5]. The MMN–consequently–does not take place when a deviating sound is presented until several exposures to the standard, because then there is yet no (or only an impoverished) memory representation (or neural model) of the standard and thus the oddball is not perceived as deviating [1], [7]. Even the most basic form of tone sequences (i.e., only one repetitive standard tone) requires several exposures of the standard sound for a neural model to be fashioned and thus elicit an MMN response when the standard tone sequence is interrupted by an oddball sound event. Furthermore, if the standard sequence changes over time, the number of exposures required to fashion a stable neural model increases as a function of the standard tone sequence’s complexity. Memory is hence tightly interwoven with attentional capture by oddball sound events [1].

### The Perceived Local Change Account of the Oddball Effect

An original explanation to the oddball effect was that a deviant involuntary captures attention merely because it has a low base rate (i.e., is rare) [8], [9]. Hence, according to this novelty account, the oddball sound elicits an attentional orienting response, that results in a behavioral interruption, because of the absence of a neural model (of the oddball). This assumption has proven to offer an insufficient explanation of the oddball effect: Manipulations of the size of the difference between the standard and the deviant–keeping the base rate of different deviants constant–have consistently shown that the magnitude of attentional capture appears to be a function of this difference, rather than the base rate of the deviant. For instance, Yago, Corral and Escera [10] manipulated the pitch (the term ‘pitch’ is used synonymously with ‘frequency’, as in Hertz, throughout this article for the sake of clarity although we acknowledge that the relationship between tone ‘pitch’ and tone ‘frequency’ is not linear) of deviant tones and found evidence to support that the magnitude of attentional capture–measured behaviorally–is a function of the difference between the pitch of the standard and that of the deviant. This finding suggests that larger discrepancies between standards and deviants could also result in a greater interruption to the focal activity measured behaviorally. Similar results have also been obtained in the spatial dimension, whereby standards have been presented in front of the participant and deviants with varying distances from the front [11], although it should be noted that this tendency for a greater effect with greater spatial distance between standard and deviant was only found for the right hand side, no effect of deviation was found to the left hand side, possibly due to hemispheric differences. In all, it seems as if the larger the difference is between the standard and the deviant, the greater the magnitude of attentional capture (see also [12]).

Taken together, these results suggest that the magnitude of attentional capture could simply be a local effect (that depends on the magnitude of the difference between the deviant and its predecessor) and have nothing to do with either novelty (i.e., base rate) or expectations. Specifically, according to this account, when a deviant tone, with a relatively low pitch (e.g., a 220 Hz tone), is embedded in a sequence of standards (e.g., 660 Hz tones), it captures attention to a larger degree than that of a deviant tone which is less different from the preceding standard (e.g., a 440 Hz tone), because the physical change (i.e., the difference between the most recent previous sound and the deviant) that is perceived when the deviant is presented, is larger when the deviant with relatively low pitch is presented.

The perceived local change account supposes that any sound will capture attention if it differs perceptually from the preceding stimulus. On this account, capture should occur *irrespective* of the frequency of occurrence (predictability) and base rate of the standards and the deviants. However, predictability (and the violation of expectation) is the basis of an alternative account of the oddball effect.

### The Expectancy-violation Account of the Oddball Effect

Another prominent explanation of the oddball effect is that the deviants capture attention because they violate expectations [13]. In support of this view, it has been shown that the repetition of a stimulus embedded in an otherwise changing sequence, such as the repetition of B in the sequence ABABABB, captures attention [14], [15]. Moreover, the “8” in the spoken sequence “1234568” captures attention whereas numbers that conform to the arithmetic pattern does not [16]. These findings point to the possibility that sound does not have to be novel, per se, to capture attention. Rather, attentional capture takes place when some rule governing the sound environment is violated [17]–[20]. Parmentier, Elsley, Andrés and Barceló [21] recently reported direct support for this account. They presented standards and deviants in a predictable pattern and found that standards have the power to capture attention, similar to deviants, if they are presented out of pattern. Hence, a sound event (whether it is a deviant or a standard) captures attention when it violates what the participants expect on the basis of a pattern or rule learned from past experience with the sound environment. If this is the case, the magnitude of the behavioral interruption, that follows when attention is captured, should be a function of how much the oddball event differs from expectation.

### The Present Study

A key difference between the perceived local change account and the expectancy-violation account is that the former account explains the oddball effect with reference to the difference between the deviant and the standard that *precedes* the deviant, whereas the latter account explains the oddball effect with reference to the difference between the deviant and the standard sound that is *replaced* by the deviant. In the typical oddball distraction paradigm, the same standard is presented on almost all trials, and when the oddball is presented, it deviates (retrospectively) from the preceding standard sound and (prospectively) from the expectation of another standard. Because of this, typical oddball studies cannot settle whether the observed magnitude of attentional capture is a function of the difference between the deviant and the preceding standard (as suggested by the perceived local change account) or rather a function of the difference between the deviant and an expectation of a sound that should have been presented instead of the deviant if the standard sound sequence would not have been interrupted by the deviant (as suggested by the expectancy-violation account). To tease the accounts apart, one has to compare two deviant sound conditions wherein the stimulus change from the preceding standard tone (e.g., a 660 Hz tone) to the deviant (e.g., a 1100 Hz tone) is the same in both conditions, but the conditions have to differ with regard to which standard tone is replaced by the deviant (e.g., a 440 Hz tone or a 880 Hz tone). Here, the perceived local change from the most recent standard to the deviant is equal in both deviant sound conditions (i.e., a change of 440 Hz) but the difference between the deviant and the replaced standard differs between conditions (i.e., either by 660 Hz or by 220 Hz).

**Figure 1:**
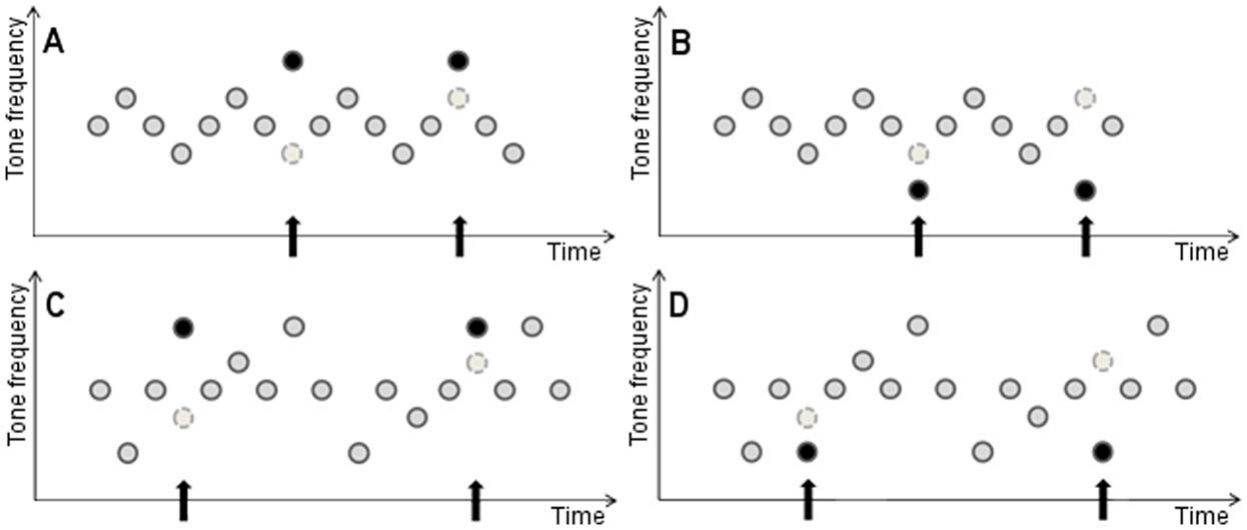
The figure shows an illustration of the four experimental conditions in Experiments 1 (panels 1A and 1B) and 2 (panels 1C and 1D), respectively. Panels 1A and 1C depict the conditions where a high-pitch deviant replaces either a high or low pitch standard. Panels 1B and 1D depict the conditions where a low-pitch deviant replaces either a high or low pitch standard. Dark grey circles represent standard tones, light grey circles represent replaced (arguably expected) tones and black circles represent replacing deviant tones.

The experiments reported here were designed to clarify whether the magnitude of attentional capture by auditory deviation is a function of the difference between the oddball sound and the standard sound that is replaced by the oddball, rather than of the difference between the oddball and the standard sound that precedes the oddball. The way this was done requires a detailed explanation (Figure 1). We borrowed a technique from previous studies on predictive auditory processing and sequence learning, whereby different tones are presented in a sequential and regular pattern (e.g., [22]; for a review see [13]), and combined this technique with the cross-modal oddball distraction paradigm. In Experiment 1, three standard sounds (a 440 Hz, a 660 Hz and an 880 Hz tone) were arranged in a repetitive cross-trial sequence (i.e., 660-880-660-440-660-880-660-440-660- etc.). During the experimental session, a sound in the standard sequence (either the 440 Hz tone or the 880 Hz tone) was occasionally exchanged with an oddball sound (either a 220 Hz tone or an 1100 Hz tone) that either differed markedly (a difference of 660 Hz) or only slightly (a difference of 220 Hz) from the sound it replaced. Most importantly, when a particular oddball is presented in this context, the change in pitch from the preceding standard sound to that particular oddball sound is held constant, but the tone that is replaced by the oddball is different between conditions: When the 220 Hz oddball is presented instead of the 440 Hz standard, the difference between the oddball and the standard is smaller than when the 220 Hz oddball replaces the 880 Hz standard; and when the 1100 Hz oddball is presented instead of the 880 Hz standard, the difference is smaller than when it replaces the 440 Hz standard.

If violated expectations underlie the magnitude of attentional capture, a cross-over interaction should be revealed. Given that the participants have sufficiently learned the standard sound sequence, the 220 Hz oddball should be more captivating when it replaces the 880 Hz standard than when it replaces the 440 Hz standard, because the difference between the replaced (or expected) tone and this particular oddball is larger in the first case. In contrast, the 1100 Hz oddball should be more captivating when it replaces the 440 Hz standard than when it replaces the 880 Hz standard for the same reason. If, however, perceived local change is the dominant determinant of attentional capture by auditory events, the oddball sounds’ capability to capture attention should not depend on the pitch of the replaced tone, as the difference between the preceding standard and the oddball is always the same. To reiterate, the perceived local change account cares not about the difference in magnitude between the presented oddball and replaced tone, only about the magnitude of the difference between the presented oddball and the preceding standard tone which, regardless of whether the oddball replaces a high pitch tone or a low pitch tone, is the same magnitude (440 Hz).

## Experiment 1

### Method

#### Participants

A total of 50 students at the University of Gävle took part in this experiment. They all reported normal hearing and normal or corrected-to-normal vision and they received a small honorarium in exchange for participation. The study was approved by the Regional Ethical Review Board at the University of Uppsala (Dnr 2011/108). As the data would be treated anonymously, and no apparent ethical research complication with participation could be identified, oral consent was deemed sufficient by the Ethical Review Board. The datacollector took note of the oral consent.

#### Materials

The oddball task was modeled after Parmentier [4]. The participants were requested to respond to arrows randomly pointing either to the left (<<<) or to the right (>>>) by pressing the corresponding arrow key on a computer keyboard. The computer recorded the response time (RT) between the onset of the arrow and when the participant pressed a button. The arrow was visible for 600 ms and was then replaced by a 250 ms visual mask (###). Any key press that took place after the arrow had disappeared was considered an error response. Each arrow was immediately preceded by a 200 ms (10 ms rise and fall time) sinewave tone. There were three standard tones, 440 Hz, 660 Hz and 880 Hz. The order of the standards were arranged in a repetitive sequence across trials (i.e., 440-660-880-660-440-660-880-660- etc.). Occasionally during the experimental session, either the 440 Hz tone or the 880 Hz tone was exchanged with either a 220 Hz oddball tone or an 1100 Hz oddball tone. The tones were normalized and presented binaurally through headphones (Sennheiser HD 202) at approximately 65 dB(A). The temporal distance between tones was 1050 ms (onset-to-onset).

#### Design and Procedure

A within-participant design was used. Participants sat alone in a silent room in front of a computer with headphones attached. The computer controlled stimulus presentation and recording of responses. In advance of testing, the participants were told to ignore all sound, to use their dominant hand when responding to the arrows, and to respond as accurately and quickly as possible. Testing the hypotheses generated by the violated-expectations account requires that the participants learn the standard tone sequence appropriately. To meet this requirement and to familiarize the participants with the task, they began with a training phase that contained a total of 60 repetitions of the 660-880-660-440 tone sequence. Since the first encounters with deviants are much more captivating than later encounters (e.g., [7], [23]), probably due to habituation or a startle response, the two oddball sounds (6 of each) were randomly interspersed in exchange of the 440 Hz and 880 Hz tones during the latter half of the training phase, to avoid noise in data due to these factors. After the training phase, the participants undertook a total of 288 trials divided across two blocks. Across the two experimental blocks, the 440 Hz tone was replaced 6 times by a 220 Hz tone (i.e., -660-220-660-880-660- etc.) and 6 times by a 1100 Hz tone (i.e., -660-1100-660-880-660- etc.), and the 880 Hz tone was replaced 6 times by the 220 Hz tone (i.e., -660-440-660-220-660- etc.) and 6 times by the 1100 Hz tone (i.e., -660-220-660-1100-660- etc.). The order of the four types of tone replacements was randomized, and each type was followed equally as often by left as by right pointing arrows. There was a 25 sec pause in the middle of the training phase, before the first experimental block and before the second experimental block.

## Results and Discussion

**Figure 2:**
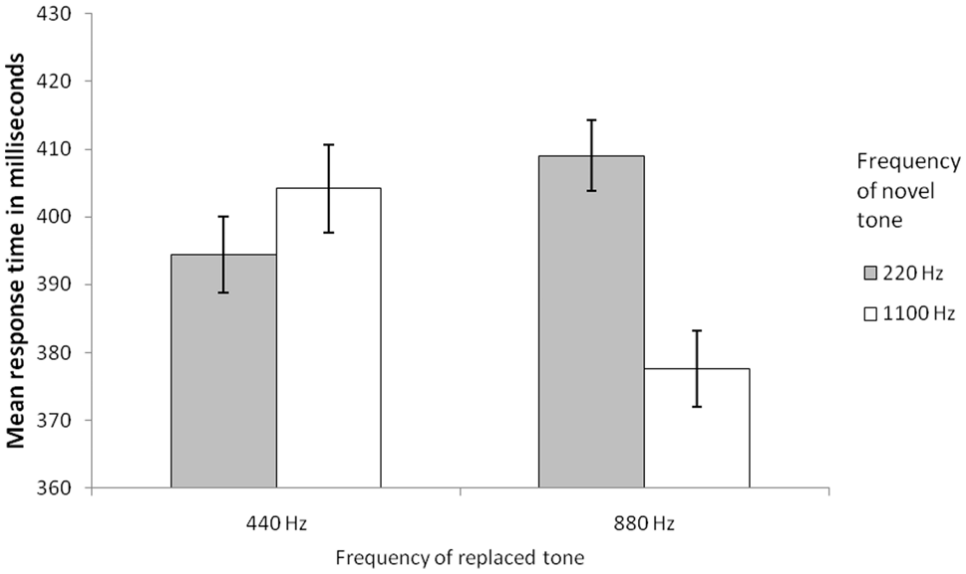
The figure shows the results from Experiment 1. Each visual target was preceded by one of three standard tones that together formed a repetitive cross-trial sequence (660 Hz –880 Hz –660 Hz –440 Hz –660 Hz –880 Hz –660 Hz –440 Hz –660 Hz etc.). Occasionally, either the 440 Hz tone or the 880 Hz tone was replaced by a 220 Hz deviant tone or an 1100 Hz deviant tone. The figure shows the mean response time to visual targets following a deviant tone for each of the four types of replacements respectively (trials with correct responses are included only). Error bars are standard error of means.

As the research question addressed here solely concerns whether an oddball sound event is–relatively–more captivating in one sound context than in another sound context, the analysis exclusively concerns trials whereby oddball sound events were presented. There were 81 error/absent responses (out of 1,200 responses in total across all participants) on oddball trials. Hence, error rate was low (<7%) and trials with incorrect responses were removed from the response time analysis. As can be seen in Figure 2, the degree to which the two oddballs captured attention was a function of the pitch of the standard they replaced. The oddball of high pitch was more captivating when it replaced a low-pitch standard, than when it replaced a high-pitch standard, whereas the oddball of low pitch was more captivating when it replaced a high-pitch standard, than when it replaced a low-pitch standard. These conclusions were statistically confirmed by a 2(Pitch of oddball tone: 220 Hz, 1100 Hz) × 2(Pitch of replaced tone: 440 Hz, 880 Hz) repeated measures analysis of variance with an alpha set to.05. This revealed a significant main effect of pitch of oddball tone, *F*(1, 49) = 5.38, *MSE* = 1082.01, *p* = .025, η_p_^2^ = .10, and a significant interaction between the factors, *F*(1, 49) = 31.28, *MSE* = 677.93, *p*<.001, η_p_^2^ = .39, but no significant main effect of pitch of replaced tone, *F*(1, 49) = 1.84, *MSE* = 968.01, *p* = .181, η_p_^2^ = .04. Pair wise comparisons confirmed that the high-pitch oddball was more captivating when it replaced a low-pitch standard than when it replaced a high-pitch standard, *t*(49) = 4.99, *p*<.001, and that the low-pitch oddball was more captivating when it replaced a high-pitch standard than when it replaced a low-pitch standard, *t*(49) = 2.39, *p* = .021.

Experiment 1 yields support for the expectancy-violation account of the oddball effect (e.g., [13]). The magnitude of attentional capture was determined by the magnitude of the difference between the encountered oddball and the replaced standard in the context of the auditory sequence. The perceived local change account fails to explain this result because it supposes that the magnitude of attentional capture is determined by the magnitude of the perceptual difference in pitch between the oddball and the preceding tone, which was the same regardless of whether a high pitch tone replaced a low or a high pitch tone or whether a low pitch tone replaced a low or a high pitch tone. Moreover, the results are also at odds with the idea that the potency of attentional capture depends entirely on the low base rate of the oddball: In Experiment 1, the base rate of the substituting oddballs were the same, only the pitch between the presented and replaced tone differed. Crucially then, Experiment 1 demonstrates that the magnitude of attentional capture is influenced by the expectancy manipulation–based on prior auditory sequence learning–even when physical changes are held constant.

## Experiment 2

The claim that Experiment 1 supports an expectancy-violation account rests on the assumption that the participants learned the standard tone sequence and, at some point, began to expect upcoming tones that would conform to the standard sequence. Moreover, if the results of Experiment 1 truly yield support for an expectancy-violation account, it should be possible to show that familiar–non novel–sounds produce the same pattern of results as the oddball sounds of Experiment 1 because, according to the expectancy-violation account, a sound captures attention to the degree it differs from a replaced sound, not because it has a low base rate. Experiment 2 was designed to explore these issues. To this end, a longer standard tone sequence was developed, which made it possible to replace low- and high-pitch tones in the standard tone sequence with other low- and high-pitch tones taken from the standard tone sequence (Figure 1).

Given the results in Experiment 1, we expected to obtain longer response times when a high-pitch tone replaced a low-pitch tone than when it replaced a high-pitch tone, and vice versa. However, due to the the extended, and thereby more complex tone sequence used in Experiment 2, the effect should emerge only towards the end of the experiment, as participants begin to expect upcoming tones on the basis of the standard tone sequence. Once the sequence is sufficiently learned, and is being used to form expectations of upcoming standards, the magnitude of attentional capture following tone replacements should be similar to those produced by low base rate sounds.

### Method

#### Participants

A total of 34 students at the University of Gävle, who did not participate in Experiment 1, took part in this experiment. They all reported normal hearing and normal or corrected-to-normal vision and they received a small honorarium in exchange for participation.

#### Materials

The oddball design was the same as in Experiment 1. The standard tone sequence used in Experiment 2 was …-660-220-660-440-660-880-660-1100-660-220-660-440-660-880-660-1100-660-220-… and so on.

#### Design and procedure

The core design was similar to Experiment 1. The major difference in Experiment 2 is the absence of novel sounds. Instead of having novels replace standard tones as in Experiment 1, the standard tones were replaced by pattern deviants constituting the exact same tones as those in the standard tone sequence (i.e., the 220 Hz tone and the 1100 Hz tone respectively). Due to the increased complexity of the standard tone sequence, the total number of trials was expanded to 816 divided across six blocks, each block consisting of 136 trials. As in Experiment 1, either the 440 Hz tone or the 880 Hz tone was replaced by a 220 Hz tone or an 1100 Hz tone. Hence, the difference between the replaced tone and the actually presented tone was either small (220 Hz) or large (660 Hz). Each type of tone replacement occurred 12 times in total (e.g., the 440 Hz tone was replaced by the 220 Hz tone 12 times) and the order of replacements was randomized within each block.

## Results and Discussion

**Figure 3:**
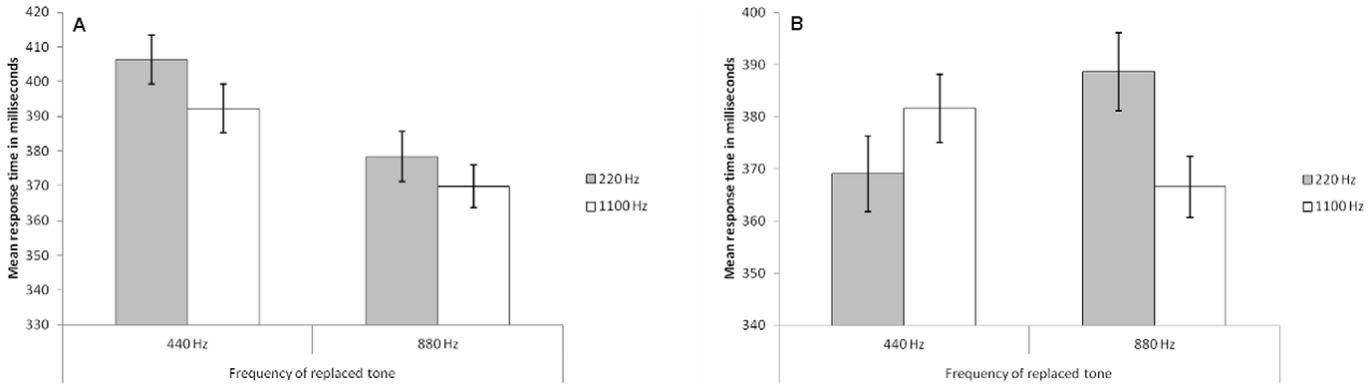
The figure shows the results from Experiment 2 divided into the first half (panel A) and the second half (panel B) of the experiment. Each visual target was preceded by one of several standard tones that together formed a repetitive cross-trial sequence (660 Hz –220 Hz –660 Hz –440 Hz –660 Hz –880 Hz –660 Hz –1100 Hz –660 Hz –220 Hz –660 Hz –440 Hz –660 Hz –880 Hz- 660 Hz –110 Hz –660 Hz etc.). Occasionally, either the 440 Hz tone or the 880 Hz tone was replaced by a 220 Hz pattern deviant or a 1100 Hz pattern deviant. The figure shows the mean response time to visual targets following a tone replacement for each of the four types of replacements respectively (trials with correct responses are included only). Error bars are standard error of means.

The analysis reported here covers only trials where tones were replaced. Incorrect responses (<8%) were excluded from the analysis. Data from trial block 1–3 was collapsed as was data from trial block 4–6 to increase the reliability of the learning analyses. As can be seen in Figure 3, high-pitch pattern deviants were more captivating when they replaced low-pitch standards than when they replaced high-pitch standards, and vice versa, at the end of the experiment (panel B), but not in the beginning of the experiment (panel A).

These conclusions were supported by a 2(Part of the experiment: Block 1–3, Block 4–6) × 2(Pitch of replaced tone: 440 Hz, 880 Hz) × 2(Pitch of presented tone: 220 Hz, 1100 Hz) repeated measures analysis of variance which revealed a main effect of pitch of replaced tone, *F*(1, 33) = 13.74, *MSE* = 650.80, *p* = .001, η_p_^2^ = .29, a significant interaction between replaced and expected tone, *F*(1, 33) = 4.78, *MSE* = 748.20, *p* = .036, η_p_^2^ = .13, and most importantly, a significant three-way interaction between the factors, *F*(1, 33) = 4.41, *MSE* = 1556.01, *p* = .043, η_p_^2^ = .12, but no significant main effect of pitch of presented tone was found, *F*(1, 33) = 4.07, *MSE* = 1076.81, *p* = .052, η_p_^2^ = .11. To further investigate whether there was any difference over the two halves of the experiment, separate analyses were conducted for the first half of the experiment (blocks 1–3) and the second half of the experiment (blocks 4–6). As can be seen in Figure 3a, there is a main effect of pitch of replaced tone, *F*(1, 33) = 27.29, *MSE* = 788.81, *p* = .007, η_p_^2^ = .45, but no main effect of pitch of presented tone, *F*(1, 33) = 3.97, *MSE* = 1093.63, *p* = .055, η_p_^2^ = .11, and no significant interaction was found between the two factors in the beginning of the experiment, *F*(1, 33) = 0.25, *MSE* = 265.44, *p* = .621, η_p_^2^ = .007. However, a clear cross-over interaction emerges in the latter part of the experiment (Figure 3b). This was confirmed by a 2(Pitch of replaced tone) × 2(Pitch of presented tone) repeated measures analysis of variance with response time from blocks 4–6 as dependent variable. The analysis revealed no main effect of replaced tone, *F*(1,33) = 0.31, *MSE* = 540.17, *p* = .58, η_p_^2^ = .009, no main effect of presented tone, *F*(1,33) = .85, *MSE* = 902.07, *p* = .362, η_p_^2^ = .03, but a significant interaction between the two factors, *F*(1,33) = 8.23, *MSE* = 1236.63, *p* = .007, η_p_^2^ = .20. Pair wise comparisons were conducted to further investigate these effects. The analysis confirmed that the 220 Hz tone was more captivating when it replaced the 880 Hz tone than when it replaced the 440 Hz tone, *t*(33) = 2.45, *p* = .020, and that the 1100 Hz tone was more captivating when it replaced the 440 Hz tone than when it replaced the 880 Hz tone, *t*(33) = 2.36, *p* = .024.

Experiment 2 conceptually replicates the results of Experiment 1: The magnitude of attentional capture seems to depend on the degree of pitch difference between the encountered and replaced tone. The new feature of this experiment, however, is that it shows that the pitch of pattern deviants, that were the exact same tones as those within the standard sequence, modulates the magnitude of attentional capture. This effect of substitution emerged in the second half of the experiment, indicating that the effect emerges first when participants sufficiently learned the repetitive standard tone sequence. With this in mind, it is interesting to note the striking similarity between the results from the latter half of Experiment 2 (Figure 3b) and what was found in Experiment 1 (Figure 2). Apparently, it took the participants longer to learn the more complex auditory sequence of Experiment 2, in contrast to the rather more simple sequence of Experiment 1. These results are consistent with the expectation-violation account as expectations develop with greater exposure to the auditory sequence. Indeed, the long build up itself counts against the perceived local change account and the novelty account.

## General Discussion

Collectively, these two experiments demonstrate that an event captures attention to the extent that it violates expectations for another event. In Experiment 1, the magnitude of attentional capture depended on the pitch difference between the deviant and replaced tone. Moreover, in Experiment 2, pattern deviants–consisting of sounds for which there is indeed recent memory–captured attention when they were presented in place of standards. Therefore, a particular sound does not capture attention to the extent that there is no recent memory for that particular sound, such as in the case when the sound has a low base rate (i.e., is “novel” [8], [16], [20]). Novelty is neither necessary nor sufficient for attentional capture (see also [21]). Importantly, the results also undermine a strong version the perceived local change account wherein attentional capture is simply a function of the magnitude of the difference between the captivating event and its *predecessor*. Perceived local change may contribute to the magnitude of attentional capture, but when the difference between the deviant sound event and its predecessor is kept constant, the magnitude of attentional capture is determined by the magnitude of the difference between the *presented event* and the *replaced event*. In all, the results reported here suggest that sound events capture attention to the extent that they violate an expectation for a replaced event, based on the learning of a preceding pattern of stimulation: The auditory sequence.

The finding that the cross-over interaction in Experiment 2 ‘kicked in’ after some time further reinforces the conclusion that violated expectations underlie the magnitude of attentional capture rather than novelty or perceived local change. This is consistent with the idea that auditory sequence learning, and hence a neural model, of the irrelevant sequence takes time to fashion, is used as an abstract forward (predictive) model of the sequence pattern and forms the basis of detection of any violations in the unfolding irrelevant stream [8], [14], [20], [24], [25]. The finding that the pattern of tones in Experiments 1 and 2, was extracted, compliments the results from previous auditory event-related potential studies that have shown preattentive pattern extractions of the relations between temporally adjacent and nonadjacent stimuli (e.g., [9]). We suppose that these rule-based expectancies are preattentively learned and that the inferences or expectancies about upcoming events/future states need not be explicit.

Our results are consistent with recent work using the visual-verbal serial recall task [20] in which attentional capture by concurrent irrelevant speech sequences resulted in poorer serial recall performance when the gender of the voice conveying the speech items changed every five recall trials, but not when the irrelevant sequence is first encountered and hence novel. In this case, attentional capture occurred first when the expectation of hearing a particular voice was violated. Of more relevance to the current experiments is the finding that the capture response diminished over the course of the experimental session as the expectation of voice change every five trials built up, but was restored when the session wide expectation itself was violated after a break in the pattern of change in voice every 5 trials. In common with the experiments reported, the attentional capture observed is consistent with the notion that a buildup is required for attentional capture and that sound, itself, does not have to be novel to capture attention.

Whilst our results support the expectancy-violation account over the perceived local change account, it should be mentioned that the expectancy-violation account has not always received unequivocal support in different settings. For example, consistent with the expectancy-violation account and at odds with the perceived local change account, Parmentier et al. [21] found that when a standard predictably followed two deviants, response times were significantly shorter than in conditions in which the sound was not predictable, even though there was a physical change from the preceding trial. However, consistent with the perceived local change account, and at odds with the expectancy-violation account, is that response times were longer when a standard, predictably, followed after two deviants (at which there is indeed physical change from the preceding sound to the standard) in comparison with standards that followed, predictably, after other standards (at which there is no physical change from one sound to the next).

Moreover, an alternative to the ‘pitch difference hypothesis’ (i.e., the assumption that the magnitude of auditory attentional capture is determined by the difference in pitch between the expected sound and the actually presented sound) needs to be outlined and discussed. In the two conditions with a large difference between the actually presented sound and the replaced sound, the direction of change of the sound sequence is reversed. That is, the pitch of the tones preceeding the replacement (e.g., 440-660- Hz) is moving in one direction (e.g., increasing pitch) and the replacement (e.g., a decrease in pitch) alters the direction of this change (e.g., 440-660-*220*- Hz). The replacement, hence, violates an expectation for the direction of pitch change, based on the standard tone sequence (e.g., 440-660-880- Hz). In contrast, in the two conditions with a small difference, the replacement (e.g., 440-660-*1100*- Hz) is consistent with the direction of change of the standard tone sequence (e.g., 440-660-880- Hz). Hence, an alternative ‘change direction hypothesis’ can be advocated to explain the results. In short, according to this simpler explanation, the reason why longer response time is observed after a greater difference between the replaced tone and the actually presented tone is because the replacement violates an expectation of the direction of change. It should be noted that both hypotheses (the ‘pitch difference hypothesis’ and the ‘change direction hypothesis’) are consistent with an expectation-violation account, they differ only on how they characterize the cognitive representation that constitutes the expectation (i.e., an exact stimulus according to the ‘pitch difference hypothesis’ versus a change dimension according to the ‘change direction hypothesis’). Yet, the ‘pitch difference hypothesis’ has a more powerful explanatory compass as it offers an explanation to why greater differences between standards and deviants, in traditional oddball paradigms, result in greater magnitude of auditory attentional capture (e.g., [10]–[12]). The ‘change direction hypothesis’ offers no explanation for this body of results.

Another target for future experiments would be to examine effects of the magnitude of the perceived difference between expected sound and deviant sound, rather than the difference in Hertz between the expected sound and the deviant sound (as done here). The discrepancy between these two types of difference could explain the main effects of deviant tone pitch and replaced tone pitch that were found here, and examining this discrepancy would also help characterize the nature of the cognitive representation that constitutes the expectation.

To conclude, the expectancy-violation account yields an adequate explanation of the full pattern of behavioral data observed in this study. The novelty explanation (lack of a neuronal model) and the perceived local change account, are both inadequate as explanations of why the magnitude of attentional capture produced by substituting tones within a learned sequence is functionally related to the magnitude of the difference between expected and actually presented tone. The violation of a structured, rule-based, neural model accounts for this effect relatively parsimoniously. Moreover, these results underscore the need to re-evaluate the results of extant studies looking at how the difference between the deviant and standard influences the magnitude of the oddball effect. The results of the present study suggest that the crucial factor determining the degree of capture may not be the difference between the oddball and the standard per se–as the perceived local change account supposes–but rather the difference between the actually presented stimulus and the expected stimulus.
